# Chokeberry Pomace as a Component Shaping the Content of Bioactive Compounds and Nutritional, Health-Promoting (Anti-Diabetic and Antioxidant) and Sensory Properties of Shortcrust Pastries Sweetened with Sucrose and Erythritol

**DOI:** 10.3390/antiox11020190

**Published:** 2022-01-19

**Authors:** Ewa Raczkowska, Paulina Nowicka, Aneta Wojdyło, Marzena Styczyńska, Zbigniew Lazar

**Affiliations:** 1 Department of Human Nutrition, Faculty of Biotechnology and Food Science, Wrocław University of Environmental and Life Sciences, 37 Chełmońskiego Street, 51-630 Wrocław, Poland; marzena.styczynska@upwr.edu.pl; 2 Department of Fruit, Vegetable and Nutraceutical Plant Technology, Faculty of Biotechnology and Food Science, Wrocław University of Environmental and Life Sciences, 37 Chełmońskiego Street, 51-630 Wrocław, Poland; paulina.nowicka@upwr.edu.pl (P.N.); aneta.wojdylo@upwr.edu.pl (A.W.); 3 Department of Biotechnology and Food Microbiology, Faculty of Biotechnology and Food Science, Wrocław University of Environmental and Life Sciences, 37 Chełmońskiego Street, 51-630 Wrocław, Poland; zbigniew.lazar@upwr.edu.pl

**Keywords:** shortcrust pastries, LC-MS, polyphenolic compounds, antioxidant activity, anti-diabetic activity, nutritional value

## Abstract

In this study, an attempt was made to develop shortcrust pastries containing different amounts of chokeberry pomace (0%, 10%, 30%, 50%), modulating their degree of sweetness via the application of sucrose or erythritol. The obtained products were assessed for their nutritional value (energy value, protein, fats, dietary fibre, sugars, minerals). Bioactive compounds, as well as antioxidant and anti-diabetic properties in an in vitro model and sensory attributes, were also analysed. Increasing the proportion of chokeberry pomace in shortcrust pastries improved their nutritional value, especially their energy value (reduction of nearly 30% for shortcrust pastries with 50% pomace sweetened with erythritol), nutritional fibre content (10-fold higher in shortcrust pastries with the highest proportion of pomace) and potassium, calcium, magnesium, and iron content. Chokeberry pomace was also a carrier of 14 bioactive compounds. The most beneficial antioxidant and anti-diabetic effect was shown for shortcrust pastries containing 50% chokeberry pomace. In addition, it was shown that the use of erythritol as a sweetener has a beneficial effect on the perception of sensory attributes. Finally, it was shown that the developed products could be excellent alternatives to traditional shortcrust pastries and, at the same time, be a good way to utilize waste from the fruit industry.

## 1. Introduction

Berries are very popular in many countries, not only because of the wide range of possibilities for their use, but mainly because of their health-promoting qualities. They are, among other things, a source of vitamins, minerals, dietary fibre, and polyphenolic compounds [1]. One of the berries is chokeberry (*Aronia melanocarpa* L.). This fruit is one of the richest sources of bioactive compounds and has high nutritional value. It contains carbohydrates, organic acids, dietary fibre, amino acids, vitamins, and minerals [2,3]. The main group of polyphenolic compounds found in chokeberry is pro-cyanidin polymers, which account for 66% of the fruit’s polyphenols. Nearly 40% of the antioxidant activity of chokeberry is attributed to these compounds [4]. Chokeberry and products resulting from its processing show strong antioxidant properties, and the high content of polyphenols has anti-inflammatory, anticancer, antibacterial, antiviral, anti-diabetic, anti-atherosclerotic, and hypotensive effects [5,6,7,8]. The anti-inflammatory activity of chokeberry fruit is primarily associated with the prevention of cardiovascular disease, diabetes, and disorders of the immune system. This fruit has also been shown to have a beneficial effect on glucose metabolism, exhibiting anti-diabetic properties [2]. It can also be used in the prevention of inflammation, due to its antibacterial and antiviral properties. Chokeberry has been shown to be effective in treating various subtypes of influenza viruses. It inhibits the multiplication of the influenza virus in the early stages of the disease [9]. The anticancer activity is mainly related to the presence of chlorogenic acid derivatives, some cyanidin glycosides, and quercetin derivatives [10]. Polyphenolic compounds have a beneficial effect on cardiovascular function by reducing vascular oxidative stress. Skoczyńska et al. [11] proved that, in men with hypercholesterolaemia, drinking chokeberry juice contributed to the reduction of serum triglycerides, total cholesterol, and LDL (Low Density Lipoprotein) fraction. Arterial blood pressure (systolic and diastolic) was also significantly reduced [11].

Chokeberry fruit is rarely used for direct consumption, due to its characteristic astringent taste resulting from the presence of polyphenols and tannins [12]. It is most often used for preparing jams, juices, syrups and tinctures, and is also consumed in dried or candied form. Increasing attention is also being paid to the use of the by-products of fruit processing, such as pomace. They represent a cheap and readily available raw material for further processing [13]. In addition, the concentration of compounds responsible for the astringent taste of chokeberry decreases during processing, so it is more acceptable to consumers. Fruit pomace after juice extraction is still a good source of, among other things, polyphenols, vitamins, and dietary fibre, and at the same time, has a low energy value [14,15]. The most common method of preserving pomace, also called marc or bagasse, is drying. Dried and powdered pomace is added to bakery products, confectionery, functional products, and teas, among other products [16,17,18,19]. Oszmiański and Wojdyło [20] also proved that the content of procyanidins in marc is higher than in juices and fresh fruit. The regular consumption of foods rich in antioxidant compounds is closely associated with a lower risk of chronic disease [20].

In recent years, the impact of nutrition on human health has increased in importance. One of the greatest challenges of modern medicine and dietetics is the increasing prevalence of overweight and obesity and their complications, especially type 2 diabetes and cardiovascular diseases. Obesity has been classified as an epidemic of the 21st century by the WHO, and current epidemiological findings are also alarming. Over the past three decades, there has been a progressive decrease in the age threshold of overweight individuals. Of particular concern is the increasing prevalence of overweight and obesity in the developmental population. The long-term health consequences of early increased body weight lead to the development of many other chronic diseases, reduced quality of life, and reduced fitness in adulthood [21]. A strategy to curb this epidemic should combine actions aimed at the entire population, aimed at disseminating desired behaviours, and targeted at selecting foods and dishes with proven benefits for human health. Bakery products, especially pastries, are considered the most cost-effective and consumer-acceptable carriers of health-promoting substances [22].

Therefore, an attempt was made to bake shortcrust pastries containing different amounts of chokeberry pomace (0, 10, 30, 50% by weight of flour). In addition, sucrose was replaced with erythritol to increase its health-promoting properties. The studied shortcrust pastries’ variants were characterized in terms of their nutritional value (energy value, protein, fats, dietary fibre, ash, sugars and minerals), and antioxidant (ABTS- free radical scavenging activity, ORAC- oxygen radical absorbance capacity) and anti-diabetic properties (α-amylase, α-glucosidase and lipase). A quantitative and qualitative analysis of bioactive compounds was also performed (UPLC-PDA-Q/TOF-MS- ultra-performance liquid chromatography photodiode detector-quadrupole/time-of-flight mass spectrometry). The sensory attributes of shortcrust pastries were also evaluated (9-point hedonic scale). This is the first comprehensive study to evaluate the numerous health-promoting, nutritional, and sensory properties of shortcrust pastries containing different amounts of chokeberry pomace, and simultaneously using erythritol as a sweetener. Nowadays, both researchers and the food industry are focusing on developing food formulations with functional characteristics, so the present study is in line with global trends.

The aim of this study is to evaluate the possibility of using chokeberry pomace as a component shaping the content of bioactive compounds, nutritional, health-promoting and sensory properties of shortcrust pastries sweetened with sucrose and erythritol.

## 2. Materials and Methods

### 2.1. Reagents and Standards

All reagents necessary for the determination of antioxidant properties (ABTS, ORAC) were purchased from Merck (Darmstadt, Germany); those for determining anti-diabetic properties (α-amylase, α-glucosidase and lipase) and dietary fibre content were purchased from Sigma-Aldrich (Steinheim, Germany). Reagents for the quantitative and qualitative analysis of polyphenolic compounds by UPLC-PDA-Q/TOF-MS and sugars by HPLC were purchased from POL-AURA (Poland). Standards of polyphenolic compounds were purchased from Extrasynthese (Lyon, France). Chemical reagents for nutritional determination were purchased from Idalia (Radom, Poland). Standards for elemental determinations were purchased from Merck (Darmstadt, Germany), those for CRM (certified reference material) from MS Spectrum (Warsaw, Poland).

### 2.2. Characteristics of Shortcrust Pastry Products Tested

Eight variations of shortcrust pastries were made. Within those for each sweetener (sucrose, erythritol), 0%, 10%, 30%, and 50% of the flour weight were replaced with chokeberry pomace (labelling of shortcrust pastries variants: SA—sucrose-sweetened shortcrust pastries with specific addition of chokeberry pomace; EA—erythritol-sweetened shortcrust pastries with specific addition of chokeberry pomace; 0, 10, 30, 50—% addition of chokeberry pomace to flour weight). Table 1 shows the raw material composition of the different shortcrust pastries’ variants. Chokeberry pomace was purchased from the certified company GreenHerb (Certificate of the Institute for Consumer Research, Łańcut, Poland).

The preparation of the shortcrust pastry started with mixing the wheat flour/chokeberry pulp with powdered sugar or erythritol. Then, the chopped butter and egg yolks were added. The dough was kneaded with a KitchenAid model 5KPM5 mixer (Springfield, OH, USA) until the ingredients were combined (3 min). A ball was formed from the dough thus prepared, wrapped in cling film, and refrigerated at 4 °C for 1 h. Then, the dough was rolled out to a thickness of 0.5 cm; circles of 5 cm diameter were cut out and baked for 8 min at 180 °C in a Rational Combi Steamer (Landsberg am Lech, Munich, Germany).

### 2.3. Nutrition Labelling

Energy value, water and dry matter content, and total ash were determined using standard AACC (American Association of Cereal Chemists) methods [23]. Protein content was determined by the Kjeldahl method [24], and fat content by the Soxhlet method [25].

The content of assimilable carbohydrates in the studied shortcrust pastries was calculated using the following equation:

assimilable carbohydrates = dry matter − (dietary fibre + fat + protein + ash)(1)

### 2.4. Determination of Sugars

Sugar content was determined by HPLC-ELSD (Merck-Hitachi L-7455; Merck KGaA, Darmstadt, Germany), with an ELSD of 1000 (Polymer Laboratories Inc., Amherst, MA, USA). This method was previously described by Wojdyło et al. [26]. To approximately 4–5 g of sample, 100 mL of distilled H_2_O was added. They were then subjected to ultrasound for 15 min (Sonic 6D; Polsonic, Warsaw, Poland) and heated for 30 min at 90 °C, with occasional stirring. After this time, each sample was centrifuged (MPW-55; Warsaw, Poland) at 19,000× *g* for 10 min, and the resulting supernatant was filtered through Sep-Pak C-18 cartridges (Waters Millipore, Milford, MA, USA) and through a 0.20 mm hydrophilic membrane PTFE filter (Millex Samplicity Filter; Merck, Darmstadt, Germany). The samples prepared in this way were used for further analyses.

The separation of sugars was performed using a Prevail™ Carbohydrate ES HPLC Column-W (250 × 4.6 mm × 5 mm; Imtakt, Kyoto, Japan). Oven temperature was set to 30 °C. The flow rate was 1 mL/min; the mobile phase was used with an acetonitrile:water mixture (75:25, *v*/*v*) for isocratic elution; and an injection volume of 20 μL. The following parameters were used: 80 °C for the nebulizer, 1.2 mL/min for the nitrogen gas flow, and 80 °C for the evaporative temperature. Calibration curves (*r*^2^ = 0.9998) of reference standards sucrose, glucose, fructose, mannitol, sorbitol and erythritol were used to quantify sugars. Standards were injected under the same conditions at concentrations of 1–10 mg/L (*r*^2^ = 0.999–0.997), presented as the sum of sugars. Data were presented as the mean of three replicates and expressed as g/100 g of cookies, with the appropriate addition of chokeberry pomace and sweetener variation.

### 2.5. Determination of Mineral Content

The content of iron, zinc and magnesium was determined using the atomic absorption spectrometry (FAAS) method. Calcium, sodium and potassium content was determined by atomic emission spectrometry (FEAS). Determinations were performed using a Varian AA240FS atomic absorption spectrometer (Mulgrave, VIC, Australia). Samples were mineralized (15 min, 210 °C) in a MARS 6 closed microwave system (CEM, Matthews, NC, USA). Approximately 0.5 g of each sample and 5 mL of nitric acid (65%) and 1 mL of hydrogen peroxide were used for digestion. The samples were then quantitatively transferred using bidistilled water into 10 mL test tubes. The accuracy of the method was confirmed using the validated reference material BCR-191 brown bread, and the measurement uncertainty was estimated to be 5% [27,28,29].

### 2.6. Quantitative and Qualitative Determination of Polyphenolic Compounds

For the qualitative and quantitative determination of polyphenolic compounds by UPLC-PDA-Q/TOF-MS and UPLC-PDA methods, the procedure was as described by Tkacz et al. [30]. About 2.00 g of each of the test cake samples and about 0.50 g of chokeberry pomace were mixed with 6 and 5 mL of methanol:water solution (3:7, *v*/*v*) with 2% ascorbic acid and 1% acetic acid, respectively, and then treated with ultrasound (Sonic-6D; Polsonic, Warsaw, Poland). The samples were stored at 4 °C for 24 h. After this time, re-extraction was performed. The samples were then centrifuged (19,000× *g*, 10 min, 4 °C; MPW-350; Warsaw, Poland). Supernatants were filtered through a hydrophilic membrane (PTFE, 0.20 µm; Millex Samplicity Filter; Merck, Germany) and used for further analysis. Phenolic compounds were analysed using an ACQUITY Ultra Performance Liquid Chromatography system (Waters Corporation, Milford, MA, USA) with a binary solvent manager and PDA detector coupled to a G2 Q/TOF micromass spectrometer (Waters, Manchester, UK), equipped with an ESI electrospray ionization source operating in positive and negative modes. UPLC-PDA-Q/TOF-MS analysis parameters were used, as previously described by Wojdyło et al. [26]. A BEH C18 UPLC column (2.1 × 100 mm, 1.7 μm; Waters Corporation, Milford, MA, USA) was maintained at 30 °C. The flow rate was 0.420 mL/min, the injection volume was 5 µL, and elution was completed in 30 min. Solvent A was 2.0% formic acid, and solvent B—100% acetonitrile. The program starts with a gradient elution from 99 to 65% by solvent A, and from 1 to 35% by solvent B for 12 min (linear), and then lowering solvent A to 0% (increase solvent B to 100%) for the condition column (12.5–13.5 min); after that, the gradient returned to the initial composition—99% of solvent A, and 1% of solvent B, until 15 min for held constant to reequilibrate the column. PDA spectra for anthocyanins, flavonols, phenolic acids and flavan-3-ols were measured at the following wavelengths: 520, 360, 320 and 280 nm, respectively. The optimized MS parameters were as follows: desolvation temperature 300 °C, source temperature 100 °C, desolvation gas flow 300 L/h, cone gas flow 40 L/h, cone voltage 30 V, and capillary voltage 2500 V. An MS analysis was performed using mass scanning from *m*/*z* 100 to 1200. Empower 3 software and MassLynx 4.0 ChromaLynx Application Manager software (Waters Corporation, Milford, Connecticut, United States) were used to process the quantitative and qualitative data. Quantitative evaluation was performed by injecting solutions of chlorogenic acid, procyanidin B1, and kaempferol-3-*O*-glucoside and -galactoside, with known concentrations ranging from 0.05 to 5 mg/mL (r^2^ ≤ 0.9998) as standards. The results of UPLC-PDA analyses were given as the mean of three replicates and expressed as mg per 1.0 kg of shortcrust pastries containing chokeberry pomace.

### 2.7. Analysis of Biological Activity

Approximately 2.5 g of shortcrust pastries and about 0.5 g of ground dry chokeberry pomace were weighed into test tubes and mixed with 7 and 8 mL of 80% aqueous methanol (acidified with 1% HCl), respectively. The resulting suspension was stirred. The samples were subjected to ultrasound twice in a Sonic 6D water bath (Polsonic, Warsaw, Poland) (15 min) and then centrifuged (5 min, 1000× *g*).

The iron-reducing capacity of 2,2′-azino-bis(3-ethylbenzothiazoline-6-sulfonic acid) (ABTS) was analysed according to Re et al. [31]. A Shimadzu UV-2401 PC spectrophotometer (Kyoto, Japan) was used for this purpose. The ORAC (oxygen radical absorbance capacity) test was performed using a Shimadzu RF-5301 PC spectrofluorometer (Kyoto, Japan), according to the guidelines previously described by Ou et al. [32]. All determinations were performed in triplicate. The results were expressed as mM Trolox per 100 g of shortcrust pastries containing chokeberry pomace.

Anti-diabetic activity (α-amylase, α-glucosidase and lipase) was determined according to the guidelines described by Nowicka et al. [33] and Podsędek et al. [34]. Each sample was analysed in triplicate, and the result was expressed as the IC50; i.e., the amount of sample capable of reducing the activity of a specific enzyme by 50%. A UV-2401 PC spectrophotometer (Shimadzu, Kyoto, Japan) was used for the analysis. p-Nitrophenyl-α-D-glucopyranoside, potato starch and p-nitrophenyl were used as the substrate.

### 2.8. Sensory Evaluation

Each variant of shortcrust pastries was evaluated for sensory attributes, i.e., colour, taste, smell, crispness, and overall acceptability, on a 9-point hedonic scale [35]. Sixty-two people participated in the study. Each of them was informed about the purpose of the study and given a questionnaire to record their own observations. The description of the scoring was as follows: 1—strongly dislike, 2—dislike a lot, 3—dislike, 4—slightly dislike, 5—neither like nor dislike, 6—slightly like, 7—like, 8—like a lot, 9—strongly like. The tests were conducted in a sensory room free of food/chemical odour, sound, and interfering light. The study was conducted in accordance with the guidelines of the Declaration of Helsinki [36]. All personal data of the participants in the organoleptic evaluation have been anonymized in accordance with the General Data Protection Regulation of the European Parliament (GDPR 679/2016).

### 2.9. Statistical Analysis

All statistical analyses and PCA were performed with Statistica version 13.3 (StatSoft^®^, Tulsa, OK, USA). Significant differences (*p* ≤ 0.05) between means were evaluated by one-way ANOVA and Tukey’s multiple range test. Spearman’s correlation coefficient was used to assess the relationship between the selected variables. All data are presented as the mean value ± standard deviation and were performed at least three times. Chokeberry pomace was not considered in the statistical analysis. Their nutritional value, polyphenolic compound content, and health-promoting properties are tabulated to present the properties of the starting matrix.

## 3. Results and Discussion

### 3.1. Nutritional Value

Table 2 presents the energy value and content of selected nutrients, ash and dry matter in 100 g of shortcrust pastries containing chokeberry pomace. As the proportion of chokeberry pomace added increased, the energy value decreased in both sucrose- and erythritol-sweetened cakes. In addition, erythritol-sweetened shortcrust pastries had a statistically significantly lower energy value than those with sucrose. The content of dietary fibre and ash depended on the cake variant—the more pomace added, the higher their concentration. Fibre content ranged from 2.79% (SA0 shortcrust pastries) to 29.67% (EA50 shortcrust pastries), while ash content ranged from 0.45% (SA0 shortcrust pastries) to 0.77% (EA50 shortcrust pastries). An increase in dietary fibre content and a decrease in energy value were also shown by Ismail et al., in their study, in which they enriched pastries with pomegranate peel [37], and by Youssef and Mousa, who used citrus peel as an additive in pastry products [38]. The ash content was significantly higher in shortcrust pastries containing 30% and 50% marc compared to traditional shortcrust pastries. This relationship applied to both sucrose- and erythritol-sweetened products. The ash content of flour, depending on the wheat variety, varies from 0.54% to 1.36% [39], so it is lower than that of chokeberry pomace (1.91%). Statistically significant changes in dry matter content were also observed—an increasing proportion of bagasse was associated with an increase in dry matter. This is consistent with the results of the study by Czaja et al., who showed that the water content of wheat flour ranges from 9.0% to 15.0% [39], so it is higher than that of chokeberry pomace (about 5.0%). In the studied products, fat came only from the addition of butter, so the fat content of the different variants of shortcrust pastries was similar. Total carbohydrates also included polyols; therefore, their content was similar in individual shortcrust pastries variants. The carbohydrate content of wheat flour is about 77.0%, so it is about 10% lower than that of chokeberry pomace [40]. Furthermore, the results of the study by Lin et al., showed that the use of erythritol as a sweetener for Danish cookies does not significantly affect the dry matter, protein, fat, and ash content [41]. For nutritional value, there are differences between sucrose-sweetened and erythritol-sweetened cookies, but for the most part, these differences are not statistically significant. These may be due to the fact that the pomace is not a homogeneous material. The results of the study showed an attractive nutritional profile of shortcrust pastries containing chokeberry pomace, especially in terms of dietary fibre content and energy value.

#### 3.1.1. Sugar Content

The sugar profile of the products was also analysed; the results are presented in Table 3. The total sugar content ranged from 13.11 g/100 g for SA0 to 17.77 g/100 g for SA50. Sucrose and erythritol had the highest proportion of all sugars, which is due to their use as sweeteners in the different cake variants. Mannitol, sorbitol, and fructose were also identified in the cakes studied, but the presence of these sugars was found only in those with pomace added. Sorbitol was found in the highest amount (4.40 g/100 g of pomace). The sorbitol content is higher than in the study of Mayer-Miebach et al., who showed that the content of this sugar in chokeberry pomace is 37.6 g/kg [42]. On the other hand, the fructose content in the studied products is lower than that obtained by other authors [42]. The difference is about 30%. The difference is most likely due to the fact that the heat treatment of chokeberry pomace at 160 °C leads to a reduction in fructose content of about 20% [43]. Sorbitol and mannitol content also increased on the addition of chokeberry pomace to shortcrust pastries. In addition to their sweetening properties, these substances are also used by food manufacturers as fillers and stabilizers. In addition, they do not take part in Maillard reactions, characteristic of bakery products. Their content in chokeberry pomace is relatively small, so it is safe for health (such an amount will not have laxative properties), and at the same time, positively influences the nutritional properties of the studied cakes, e.g., energy value (1 g of sorbitol is about 2.6 kcal, while mannitol is 1.6 kcal) [44].

#### 3.1.2. Mineral Content per 100 g of Shortcrust Pastries Containing Chokeberry Pomace

Table 4 shows the content of selected mineral components in the studied shortcrust pastries. Among macroelements, potassium, calcium, and magnesium were found in their highest amounts (their content increased significantly with an increase in marc addition: 68.13–96.43, 40.66–110.58 and 19.81–25.77 mg/100 g, respectively). Iron was the dominant microelement. Its content also increased significantly with an increase in marc addition, and ranged from 1.28 to 9.53 mg/100 g. Lazar et al., also showed that potassium, calcium, magnesium, and iron are the dominant minerals in chokeberry pomace, but reported values different to those obtained in the present study: 294.04, 119.94–133.29, 80.35–92.77 and 13.55–18.30 mg/100 g, respectively [45]. As shown by the studies of other authors, the content of mineral components in chokeberry pomace varies. It may depend, among other things, on the type of fruit cultivation (conventional versus organic), or on the method of pomace drying and storage conditions [45,46]. Bredaliol et al., 2020 [47] showed that the higher the baking temperature, the lower the macro mineral content of the bread. Therefore, in the case of minerals, their content in the marc will also not be the same as the corresponding amount in the different cake variants. In addition, minerals, besides chokeberry pomace, are also found in other foods included in the cakes studied (wheat flour, egg yolk). Phytic acid is also present in wheat flour, which can bind Fe, Zn, Ca, and Mg, for example. The use of different sweeteners had no significant effect (*p* < 0.05) on mineral content.

### 3.2. Identification of Polyphenolic Compounds

Table 5 shows the detailed identification of polyphenolic compounds carried out by UPLC-PDA-Q/TOF-MS. An LC-MS analysis of the investigated variants of shortcrust pastries with the addition of chokeberry pomace, and only chokeberry pomace, showed the presence of 14 polyphenolic compounds, belonging to four groups of bioactive compounds, i.e., anthocyanins, flavonols, phenolic acids and flavan-3-ols (in the form of monomers and dimers). The first fraction identified in the products was anthocyanins. Four compounds were identified in this group—cyanidin-3-*O*-galactoside (*t*_R_ = 4.241 min), cyanidin-3-*O*-glucoside (*t*_R_ = 4.530 min), cyanidin-3-*O*-arabinoside (*t*_R_ = 4.754 min) and cyanidin-3-*O*-xyloside (*t*_R_ = 5.419 min), all with [M − H]^−^ at *m*/*z* = 287+. Cyanidin-3-O-galactoside, and cyanidin-3-O-arabinoside were detected as the main anthocyanins compounds in all products with the addition of chokeberry pomace, and the increase of chokeberry addition significantly influenced the final concentration of these compounds in shortcrust pastries. The identified anthocyanins are consistent with the results of studies by other authors analysing the anthocyanin profile in fruit, juice, and chokeberry pomace [7,46,47,48,49].

The pomace of chokeberry was also a carrier of flavonols. Therefore, in shortcrust pastries without their addition, this group of compounds was not identified. In turn, the addition of pomace in only 10% resulted in their presence. Flavonols were represented by six compounds, quercetin derivatives, quercetin-3-*O*-robinobioside and quercetin-3-*O*-rutinoside (*m*/*z* = 609, with a fragment at *m*/*z* = 301), and quercetin-3-*O*-galactoside and quercetin-3-*O*-glucoside at *m*/*z* = 463. Eriodictyol-glucuronide (*t*_R_ = 6.892 min) was also identified and is responsible for the characteristic sour taste of chokeberry [50]. The results obtained were consistent with data published by, among others, Oszmiański and Lachowicz [7], Oszmiański and Wojdyło [20], and Slimestad et al. [51].

The third group of compounds identified in products with chokeberry pomace are phenolic acids (caffeoylquinic acid derivatives). All of them were characterized by [M − H]^−^ at *m*/*z* 353, but with different retention times that identified them as: neochlorogenic acid (*t*_R_ = 2.983 min), cryptochlorogenic acid (*t*_R_ = 3.727 min) and chlorogenic acid (*t*_R_ = 3.968 min). Similar profiles were determined by Oszmiański and Lachowicz [7] and Lee et al. [52]. Meanwhile, the last group of polyphenolic compounds is flavan-3-ols, represented by procyanidin B2 (*t*_R_ = 4.989 min). This association has also been identified by other authors [7,20]. It should be emphasized that, as in the case of the previously discussed compounds (anthocyanins, phenolic acids, and flavonols), flavan-3-ols were identified only in products with the addition of chokeberry. This proves that pomace, as a waste material, can be used again as a donor of polyphenolic compounds, effectively enriching the chemical composition of sweet snacks.

### 3.3. Content of Polyphenolic Compounds

Table 6 shows the results for the evaluation of polyphenolic compounds in the studied samples. Anthocyanins accounted for the highest proportion of total polyphenol content in each shortcrust pastries variant (45.9% on average; from 65.43 mg/kg for EA10 to 356.76 mg/kg for EA10). Phenolic acids accounted for 31.0% of the total polyphenolic content (from 36.29 mg/kg for EA10 to 276.88 mg/kg for SA50), while flavan-3-ols and flavonols averaged 13.4% (from 12.95 mg/kg for EA10 to 117.85 mg/kg for EA50) and 9.7% (from 12.35 mg/kg for EA10 to 85.45 mg/kg for SA50), respectively. The highest content of polyphenolic compounds was observed in shortcrust pastries containing the most bagasse (50%), both in products sweetened with sucrose and erythritol (about six times more polyphenolic compounds compared to shortcrust pastries with 10% addition of bagasse). Generally, the samples analysed were statistically significantly different in terms of the content of individual polyphenolic compounds. The most abundant compound from the anthocyanin group, both in marc and individual variants of cakes, was cyanidin-3-*O*-galactoside (from 44.72 mg/kg for EA10 to 238.13 mg/kg for EA50). Similar results were obtained in the study of Rodríguez-Werner et al., which analysed, among other things, the content of polyphenolic compounds in products obtained from chokeberry—fruit, juice, and pomace [53]. The content of other anthocyanins was as follows: cyanidin-3-*O*-arabinoside > cyanidin-3-*O*-xyloside > cyanidin-3-*O*-glucoside (Table 6). Different results were obtained by Rodríguez-Werner et al., who reported that the cyanidin-3-*O*-glucoside content was higher than that of cyanidin-3-*O*-xyloside, but the difference between these compounds was small; only 6.4 mg/100 g dry weight [53]. The results of the study by Mayer-Miebach et al., confirmed that the proportion of anthocyanins in chokeberry pomace is higher than in the fruit itself (11.9–19.5 and 6.2–6.7 g/kg, respectively), while the content of procyanidins in pomace is lower than that in chokeberry fruit [54].

Another group of polyphenols identified was flavonols. Their content in individual shortcrust pastries variants increased on increasing the proportion of pomace, and ranged from 12.35 mg/kg cake (EA10) to 85.45 mg/kg cake (SA50). There were no significant differences between sucrose-sweetened and erythritol-sweetened shortcrust pastries containing the same proportion of pomace (Table 6). The content of particular flavonols in cakes and marc was as follows: quercetin-3-*O*-glucoside > derivative of quercetin > quercetin-3-*O*-rutinoside > quercetin-3-*O*-robinobioside > quercetin-3-*O*-galactoside. The flavonol content of pomace obtained by Rodríguez-Werner et al., was different from that obtained in our study (quercetin-3-*O*-rutinoside and quercetin-3-*O*-galactoside > quercetin-3-*O*-glucoside > quercetin-3-*O*-robinobioside). Quercetin-dihexoside and quercetin-3-*O*-vicianoside were also identified [53]. The content of polyphenolic compounds in chokeberry and chokeberry products depends on, among other factors, the variety of the fruit, so there may be differences in the content of individual compounds [54].

Another group of compounds identified in the studied shortcrust pastries was phenolic acids, among which chlorogenic acid was dominant, accounting for more than 70% of all compounds belonging to this group (from 26.35 mg/kg for EA10 to 202.43 mg/kg for SA50). The addition of marc significantly increased the content of this compound in particular variants of shortcrust pastries. On the other hand, cryptochlorogenic acid was present in the smallest amount—as in the case of the other compounds—the smallest amount being found in the cakes containing 10% pomace (0.25 mg/kg) and the largest in the shortcrust pastries with 50% pomace (1.85 mg/kg) (Table 6). Oszmiański and Lachowicz demonstrated a higher content of both chlorogenic acid and cryptochlorogenic acid in the chokeberry pomace powders that they tested: 848.17–1192.69 and 41.57–53.60 mg/100 g dm, respectively [7]. Rodríguez-Werner et al., however, reported a higher chlorogenic acid content in chokeberry pomace (219.00 mg/100 g dm), and found cryptochlorogenic acid to be below the detection limit [53]. The results of the study show that pomace powder obtained from uncrushed fruit is characterized by a higher content of phenolic acids compared to the same product obtained from crushed fruit. The discrepancies may be due to differences in the process of obtaining the juice, and at the same time, the chokeberry pomace, because phenolic acids undergo rapid oxidation in enzymatic reactions [7]. The last group of polyphenolic compounds found in shortcrust pastries containing varied amounts of chokeberry pomace was flavan-3-ols. The total content of flavan-3-ols in chokeberry pomace obtained in this study (Table 6) was higher than in that by Oszmiański and Lachowicz [7] and amounted to 13.4% and 1.3% of all polyphenolic compounds, respectively. In both studies, the representatives of this group of polyphenols were procyanidin B2 and eriodictyol-glucuronide. The polyphenolic compound content of cakes with a specific addition of chokeberry pomace will not be the same as the corresponding amount of pomace. This is due, among other things, to the fact that the cookies are subjected to high temperatures (180 °C). Blanch and del Castillo’s study [55] showed that increasing the temperature to 180 °C resulted in a statistically significant decrease in polyphenolic compounds. Increasing temperature causes the thermal degradation of anthocyanins. This is also confirmed by the results of Eliasov et al., study [56]. So far, shortcrust pastries containing chokeberry pomace have not been studied, so it is not possible to directly compare the results obtained with those of other authors.

### 3.4. Biological Activity

#### 3.4.1. Determination of Antioxidant Capacity by ABTS and ORAC Methods

The antioxidant activity of chokeberry pomace and shortcrust pastries prepared with it were determined using ABTS and ORAC tests (Table 7). The antioxidant activity determined by both methods increased on increasing the proportion of pomace in shortcrust pastries. The antioxidant activity determined by the ABTS method ranged from 0.02 mmoL TE/100 g (EA0) to 2.91 mmoL TE/100 g (EA50), while for the ORAC test, it ranged from 1.14 mmoL TE/100 g (EA0) to 6.39 mmoL TE/100 g (EA50). No effect of sweetener on the antioxidant properties of shortcrust pastries containing the same amount of pomace was observed. The exceptions were shortcrust pastries with 10 and 30% added pomace (ORAC) and 30% (ABTS)—antioxidant activity was significantly higher (*p* < 0.05) in sucrose-sweetened shortcrust pastries. The increase in antioxidant activity with the increasing addition of chokeberry pomace was most likely due to the production of Maillard reaction products by high temperature [57]. Additionally, sucrose participates in the Maillard reaction (unlike erythritol), so the antioxidant activity of sucrose-sweetened cookies is higher compared to erythritol-sweetened cookies. The addition of 30% and 50% marc significantly increased the free radical scavenging capacity of both sucrose- and erythritol-sweetened products. On the other hand, in the case of the ORAC test, even the addition of 10% had a significantly beneficial effect on the capacity to absorb oxygen radicals. Kapci et al., also determined the antioxidant activity of chokeberry pomace by the ABTS method, obtaining 49.6 g/kg [58]. It was also observed that, as the total content of polyphenolic compounds increased, the antioxidant activity (ABTS, ORAC) increased (Table 6 and Table 7). Analysing the relationship between the content of particular polyphenolic compounds and antioxidant activity, it can be concluded that there is a strong positive correlation between each polyphenolic compound identified and antioxidant activity determined by the ABTS method. This is confirmed by the calculated Spearman correlation coefficient which, depending on the fraction of polyphenols, ranged from r = 0.8933 (quercetin-3-*O*-galactoside) to r = 0.9384 (chlorogenic acid). In the case of antioxidant activity determined by the ORAC method, the correlations were not statistically significant. Banach et al., showed that dry chokeberry extract (but only of one batch), which was characterized by a lower content of anthocyanins and polyphenols, simultaneously showed higher antioxidant activity. The explanation of such results requires a further detailed analysis of the other components of the extract [59]. In addition, Oszmiański and Wojdyło, who studied, among other parameters, the antioxidant activity of chokeberry and its products, showed that chokeberry pomace is characterized by a much higher antioxidant activity than chokeberry fruit or juice: the antioxidant activity (ABTS) of chokeberry fruit was 439.49 μM Trolox/100 g dried weight, and that of juice was 314.05 μM Trolox/100 g dried weight, while that of pomace was 779.58 μM Trolox/100 g dried weight [20]. These results show that chokeberry pomace is still characterized by a high content of polyphenols with high antioxidant activity, and therefore is a valuable product that should be used in designing recipes for products with functional properties.

#### 3.4.2. Analysis of α-Amylase, α-Glucosidase and Lipase Inhibition Assays

We also investigated the ability of the shortcrust pastries containing increasing proportions of chokeberry pomace to inhibit the enzymes α-amylase, α-glucosidase and lipase (Table 7). All the extracts obtained from the shortcrust pastries were tested at different concentrations to investigate their inhibitory ability, which allowed the calculation of IC50 values (mg/mL). The α-amylase inhibitory effect ranged from <0.50 mg/mL (SA50, EA30, EA50) to 221.76 mg/mL (SA0). The inhibition ability of the shortcrust pastries without pomace was more than 400 times lower than that of the cakes with 50% pomace. There were no statistically significant differences between SA50, SA30, EA50 and EA30—their ability to inhibit α-amylase was the highest. In other cases, decreasing the proportion of pomace significantly increased the IC50 (enzyme inhibition capacity decreased). In addition, the use of erythritol as a sweetener had a beneficial effect on increasing the ability to inhibit α-amylase. The polyphenols that had the greatest effect on the ability to inhibit α-amylase were cyanidin-3-*O*-galactoside (r = −0.8700), cyanidin-3-*O*-glucoside (r = −0.8602), cyanidin-3-*O*-arabinoside (r = −0.8700), cyanidin-3-*O*-xyloside (r = −0.8602), quercetin-3-*O*-galactoside (r = −0.8709) and eriodictyol-glucuronide (r = −0.8602). In the case of α-glucosidase, the enzyme inhibitory capacity increased on the addition of an increasing proportion of marc to cakes. Compounds that had the greatest effect on increasing α-glucosidase inhibitory capacity were quercetin-robinobioside (r = −0.9214), quercetin-3-*O*-rutinoside (r = −0.8948), neochlorogenic acid (r = −0.8948), cryptochlorogenic acid (r = −0.9412), chlorogenic acid (r = −0.9125), and procyanidin B2 (r = −0.9391).

Pancreatic lipase is the most important lipolytic enzyme of the gastrointestinal tract. It is responsible for the hydrolysis of dietary triglycerides to monoglycerides and fatty acids, allowing the absorption of fatty foods. The inhibition of pancreatic lipase plays an important role in the prevention and treatment of obesity because it significantly reduces fat digestion, thereby reducing fat accumulation in adipocytes [60]. Lipase inhibitory activity ranged from 9.12 mg/mL (SA50) to 16.76 mg/mL (EA0); as with the other enzymes, the differences between the cake variants were statistically significant. The polyphenolic compounds which had the greatest influence on the IC50 value were: quercetin-3-*O*-rutinoside (r = −0.8417), derivative of quercetin (r = −0.8505), neochlorogenic acid (r = −0.8417), cryptochlorogenic acid (r = −0.8346), chlorogenic acid (r = −0.8505) and eriodictyol-glucuronide (r = −0.8328).

The presented study shows that the main determinant shaping the final antidiabetic activity of the tested shortcrust pastries was the addition of chokeberries pomace. Along with the increase of the amount the chokeberries pomace in pastries, their effectiveness in relation to the inhibition effect against α-amylase, α-glucoside and pancreatic lipase increased. As indicated above, the main factors of these activities were the bioactive compounds contained in chokeberry, including anthocyanins, phenolic acids, and flavonols. This relationship is also confirmed by previous studies, which showed a positive correlation between antidiabetic properties and the content of polyphenolic compounds [33,61]. However, the addition of sugars and polyols is not without significance. As can be seen, erythritol was more effective in relation to α-amylase, while sucrose supported the inhibitory effect in relation to α-glucosidase, and pancreatic lipase. The mechanism by which these substances inhibit amylase, glucosidase, and pancreatic lipase is unclear. It may be the synergy effect of many compounds, their complexation, and precipitation. Differences in the antidiabetic, and antiobesity activities of shortcrust pastries with a different addition of sweeteners may also be the result of the Maillard reaction. This is confirmed by the observations of Turkiewicz and al. [62], who showed a strong positive correlation between the ability to inhibit glucosidase and the content of 5-hydroxymethylfurfural (5-HMF)—the product of Maillard reaction. As mentioned earlier, sucrose participates in the Maillard reaction (as opposed to erythritol), so the inhibition effect against digestive enzymes of sucrose-sweetened shortcrust pastries could be higher than erythritol-sweetened shortcrust pastries, due to the formation of complexes of this reaction.

Numerous polyphenolic compounds present in chokeberry, especially anthocyanins, may exert beneficial effects, reducing high blood glucose levels by inhibiting α-glucosidase and α-amylase activity. This action reduces the risk of diabetes and plays a positive role in its treatment [63,64]. Worszytnowicz et al., conducted a study to evaluate the in vitro ability of chokeberry extracts to inhibit α-amylase and pancreatic lipase activity. Methanolic, aqueous and acetic extracts of chokeberry have been shown to inhibit α-amylase and lipase, which may indicate a role for chokeberry in obesity prevention [63]. α-Glucosidase is the enzyme responsible for the hydrolysis of oligosaccharides to monosaccharides. α-Glucosidase inhibitors are recommended for the treatment of diabetes because they can significantly delay intestinal glucose absorption, thereby reducing postprandial hyperglycaemia [65]. The results of our own studies also confirm the beneficial effect of polyphenolic compounds, especially anthocyanins, on the inhibition of α-amylase, α-glucosidase and lipase. Chokeberry pomace cookies can help improve blood glycaemic parameters, thus providing an effective means for the prevention and treatment of diabetes. The consumption of chokeberry products has been shown to reduce glycated haemoglobin and fasting glucose levels. They also affect the reduction of insulin resistance by increasing plasma adiponectin levels [66,67]. In addition, the consumption of dietary fiber, which is found in large quantities in chokeberry pomace, reduces the risk of diabetes and ischaemic heart disease, and decreases insulin resistance [68,69]. Moreover, the use of erythritol as a substitute for sucrose may support the treatment of diabetes by controlling postprandial blood glucose levels [65].

### 3.5. Sensory Evaluation

**Figure 1:**
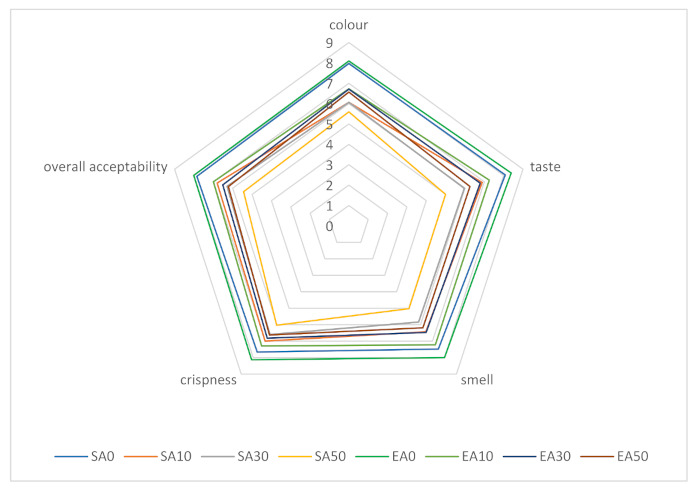
Organoleptic evaluation of shortcrust pastries containing chokeberry pomace; designation of cake variants as in Table 2.

Figure 1 shows the sensory analysis profiles of shortcrust pastries containing varying proportions of chokeberry pomace, sweetened with sucrose and erythritol. A 9-point hedonic scale was used for distinguishing factors such as colour, flavour, aroma, crispness, and overall acceptability. The highest level of acceptance in the overall evaluation of shortcrust pastries was shown for traditional shortcrust pastries (without addition of marc). It was also observed that the replacement of sucrose with erythritol had a positive effect on each of the sensory attributes, regardless of the amount of chokeberry pomace added. Increasing the proportion of pomace added decreased the acceptance of each of the differentiators, but the shortcrust pastries still had relatively high scores (5.0–7.0 points depending on the differentiators and proportion of pomace for cookies sweetened with sucrose, and 6.2–7.3 for cookies sweetened with erythritol). The addition of pomace had the least effect on brittleness evaluation. The effects of enriching shortcrust pastries with fruit pomace were also evaluated by other authors. Tańska et al., showed that the highest level of acceptance in sensory evaluation was obtained for shortcrust pastries containing rosehip pomace, while the lowest was obtained for those with rowan pomace. Regardless of the type of marc, its addition caused a decrease in the aroma evaluation of the finished products. In that study, only 20% of the flour weight was replaced by fruit pomace [70]. The results of other authors’ studies showed that the most beneficial, in terms of sensory properties, is 20% addition of strawberry marc [71]. In our study, it was shown that the use of erythritol positively influenced the sensory evaluation of the products. The results of the study by Lin et al., led to different conclusions: the addition of 75% or 100% erythritol to Danish cookies adversely affected sensory evaluation, while the addition of 25% and 50% erythritol had no significant effect [41]. The differences obtained in the two studies may be due to the addition of erythritol inducing a feeling of coolness and refreshment; therefore, it may mask the characteristic astringent taste of chokeberry, thus influencing the more favourable results of organoleptic evaluation.

### 3.6. Principal Component Analysis (PCA)

**Figure 2:**
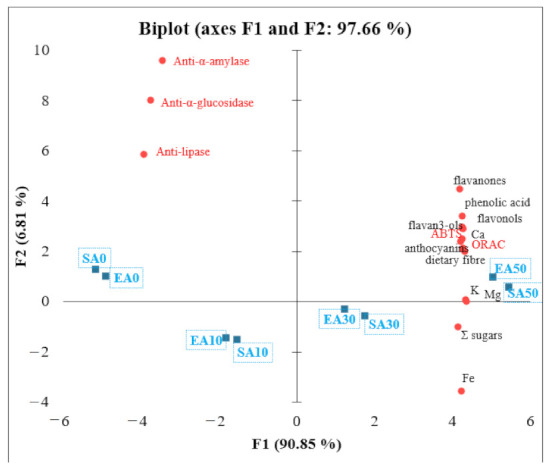
Principal component analysis (PCA)—relationships between polyphenolic compound content, antioxidant activity, anti-diabetic activity, and nutritional value.

PCA was performed to better understand the relationships between individual parameters of the chemical composition of short crust pastries containing chokeberry pomace. Factor 1 explains 90.85% of the variance, and factor 2 explains 6.81% of the variance (Figure 2). The variables that most influenced the principal component are labelled as the longest vectors in the graph. The angle between the vectors reflects the correlations between the variables. There is a negative correlation between Σ sugars and iron (Fe) and anti-α-amylase, pancreatic lipase, and anti-α-glucosidase. Flavon-3-ols, ABTS, phenolic acids, anthocyanins, ORAC, dietary fibre, potassium (K), magnesium (Mg), and calcium (Ca) have a high positive correlation. Meanwhile, flavon-3-ols, ABTS, phenolic acids, anthocyanins, ORAC, dietary fibre, potassium (K), magnesium (Mg), and calcium (Ca) correlate with anti-α-amylase, pancreatic lipase and anti-α-glucosidase. Products SA0, EA0 SA10, and EA10 present the negative correlation with anti-diabetic and anti-obesity activity. Products such as EA50 and SA50 present the high content of polyphenol compounds and antioxidant activity as ABTS and ORAC. Additionally, they present high potential to anti-diabetic and anti-obesity activity. The products of EA30 and SA30 present higher Σ sugars and iron (Fe) content and are still rich in potassium (K) and magnesium (Mg).

## 4. Conclusions

The processing of aronia into juice results in by-products, called pomace. This is an underutilized product and often discarded. On the basis of this study, it was shown that chokeberry pomace can be an excellent addition to sweet snack products, accepted by potential consumers. Increasing the proportion of chokeberry pomace in shortcrust pastries resulted in a decrease in energy value (by about 10% for SA50 biscuits compared to the traditional product and by nearly 30% for EA50) and a significant increase in dietary fibre (10-fold higher content in SA50 and EA50 compared to the traditional product), as well as potassium, calcium, magnesium and iron. It was also shown that chokeberry pomace increased the content of polyphenolic compounds, antioxidant potential, and anti-diabetic activity. According to the participants in the sensory evaluation, the use of erythritol as a sweetener had a beneficial effect on the individual parameters of the shortcrust pastries, and at the same time, contributed to a more favourable nutritional profile of the studied products. However, in the organoleptic evaluation, the overall acceptability of cakes with 50% addition of chokeberry pomace scored the least—for SA50-5.44, for EA50-6.23. In terms of consumer acceptance and at the same time, high pro-health values, it is best to add up to 30% chokeberry pomace (total content of polyphenolic compounds is then about 400 mg/kg). The antioxidant activity of 30% shortcrust pastries is also very high—70 times higher (ABTS) or 4 times higher (ORAC) than that of traditional shortcrust pastries. The anti-diabetic properties are also maintained at a very high level. The products proposed in this study could be a perfect sweet snack for people struggling with carbohydrate metabolism disorders and for those who care about their health. They could also be a good way to manage unnecessary waste in the form of fruit pomace.

## Figures and Tables

**Table 1:** Raw material composition of individual variants of shortcrust pastries.

Raw Material Composition (%)	Addition of Chokeberry Pomace			
	0%	10%	30%	50%
Wheat flour type 450	45.4	40.9	31.8	22.7
Chokeberry pomace	0.0	4.5	13.6	22.7
Butter (82% fat)	30.3			
Egg yolks	9.1			
Sugar/erythritol	15.2			

**Table 2:** Energy value and content of selected nutrients, ash, and dry matter in 100 g of shortcrust pastries containing chokeberry pomace.

Cake Variant	Energy Value (kcal)	Dry Matter (g)	Ash (g)	Fat (g)	Protein (g)	Total Carbohydrates (g)	Dietary Fibre (g)
SA0	519.7 ± 5.2 ^a^	97.51 ± 0.03 ^d^	0.45 ± 0.02 ^d^	31.16 ± 0.07 ^a^	8.41 ± 0.07 ^a^	57.51 ± 0.05 ^b^	2.79 ± 0.14 ^f^
SA10	487.4 ± 3.5 ^b^	97.70 ± 0.07 ^c^	0.52 ± 0.00 ^c^	31.43 ± 0.09 ^a^	7.59 ± 0.35 ^b^	58.20 ± 0.29 ^a,b^	8.22 ± 0.21 ^e^
SA30	482.1 ± 1.2 ^b^	98.04 ± 0.03 ^b^	0.67 ± 0.00 ^b^	32.47 ± 0.18 ^a^	6.98 ± 0.00 ^c^	57.92 ± 0.22 ^a,b^	18.06 ± 0.55 ^d^
SA50	479.3 ± 15.3 ^b^	98.33 ± 0.03 ^a^	0.78 ± 0.00 ^a^	32.14 ± 0.06 ^a^	6.29 ± 0.24 ^d,e^	59.12 ± 0.26 ^a^	28.27 ± 0.33 ^b^
EA0	426.3 ± 11.4 ^c^	93.43 ± 0.03 ^h^	0.44 ± 0.01 ^d^	29.51 ± 1.68 ^b^	6.81 ± 0.24 ^c,d^	56.66 ± 1.48 ^b^	3.17 ± 0.03 ^f^
EA10	408.2 ± 4.0 ^c,d^	96.04 ± 0.04 ^f^	0.52 ± 0.02 ^c^	31.62 ± 0.04 ^a^	6.81 ± 0.24 ^c,d^	57.10 ± 0.35 ^b^	8.90 ± 0.01 ^e^
EA30	391.1 ± 8.8 ^d,e^	96.43 ± 0.06 ^e^	0.67 ± 0.01 ^b^	32.11 ± 0.14 ^a^	4.64 ± 0.26 ^f^	58.99 ± 0.15 ^a^	18.96 ± 0.41 ^c^
EA50	382.3 ± 11.7 ^e^	95.59 ± 0.02 ^g^	0.77 ± 0.01 ^a^	31.70 ± 0.02 ^a^	5.85 ± 0.37 ^e^	57.28 ± 0.32 ^b^	29.67 ± 0.03 ^a^
WA	112.3 ± 1.6	94.84 ± 0.03	1.91 ± 0.07	0.00 ± 0.00	6.12 ± 0.00	86.80 ± 0.10	69.72 ± 0.18

All data are the mean of three measurements ± standard deviation. Values followed by the same letter within a column are not significantly different (*p* < 0.05; Tukey’s test); SA—sucrose-sweetened shortcrust pastries with specific addition of chokeberry pomace; EA—erythritol-sweetened shortcrust pastries with specific addition of chokeberry pomace; 0, 10, 30, 50—% addition of chokeberry pomace to flour weight; WA—chokeberry pomace; WA were not considered in the statistical analysis.

**Table 3:** Sugar content per 100 g of shortcrust pastries containing chokeberry pomace.

Cake Variant	Sugars (g/100 g)						
	Mannitol	Sorbitol	Glucose	Fructose	Sucrose	Erythritol	Total Sugars
SA0	nd	nd	0.10 ± 0.04 ^c,d^	nd	13.01 ± 0.25 ^b^	nd	13.11 ± 0.29 ^c^
SA10	0.09 ± 0.03 ^c^	0.06 ± 0.00 ^c^	0.21 ± 0.05 ^c^	0.10 ± 0.01 ^b,c^	14.02 ± 0.21 ^a,b^	nd	14.48 ± 0.33 ^b,c^
SA30	0.28 ± 0.02 ^a,b^	0.52 ± 0.07 ^b^	0.73 ± 0.08 ^a,b^	0.25 ± 0.02 ^b^	13.71 ± 0.65 ^a,b^	nd	15.49 ± 0.56 ^a,b^
SA50	0.42 ± 0.03 ^a^	1.02 ± 0.04 ^a^	0.89 ± 0.08 ^a^	0.62 ± 0.04 ^a^	14.82 ± 0.06 ^a^	nd	17.77 ± 0.24 ^a^
EA0	nd	nd	nd	nd	nd	13.59 ± 0.30 ^a^	13.59 ± 0.30 ^b,c^
EA10	0.11 ± 0.05 ^b,c^	0.09 ± 0.01 ^c^	0.15 ± 0.01 ^c,d^	0.09 ± 0.01 ^b,c^	nd	14.51 ± 0.06 ^a^	14.95 ± 0.02 ^a,b,c^
EA30	0.29 ± 0.02 ^a^	0.52 ± 0.11 ^b^	0.55 ± 0.05 ^b^	0.22 ± 0.03 ^c^	nd	13.80 ± 0.88 ^a^	15.38 ± 0.60 ^a,b^
EA50	0.39 ± 0.01 ^a^	0.92 ± 0.20 ^a^	0.75 ± 0.03 ^a^	0.54 ± 0.02 ^a^	nd	13.51 ± 0.97 ^a^	16.11 ± 0.68 ^a,b^
WA	2.23 ± 0.16	4.40 ± 0.32	2.53 ± 0.08	1.65 ± 0.10	nd	nd	10.81 ± 0.26

All data are the mean of three measurements ± standard deviation. Values followed by the same letter within a column are not significantly different (*p* < 0.05; Tukey’s test); designation of cake variants as in Table 2; nd—not detected; WA were not considered in the statistical analysis.

**Table 4:** Mineral content per 100 g of shortcrust pastries containing chokeberry pomace.

Cake Variant	Cu (mg/100 g)	Mg (mg/100 g)	Mn (mg/100 g)	Fe (mg/100 g)	Zn (mg/100 g)	Ca (mg/100 g)	Na (mg/100 g)	K (mg/100 g)
SA0	0.19 ± 0.05 ^b^	19.81 ± 0.28 ^d^	0.20 ± 0.01 ^d^	1.70 ± 0.03 ^e^	0.74 ± 0.03 ^b,c^	42.08 ± 2.21 ^e^	13.13 ± 0.51 ^d^	68.13 ± 1.63 ^e^
SA10	0.29 ± 0.04 ^a^	21.90 ± 0.45 ^c^	0.26 ± 0.01 ^c^	5.71 ± 0.30 ^d^	0.76 ± 0.01 ^a,b^	54.12 ± 2.56 ^d^	15.72 ± 0.24 ^b,c^	79.64 ± 1.45 ^c^
SA30	0.33 ± 0.01 ^a^	23.88 ± 0.69 ^b^	0.30 ± 0.01 ^c^	6.92 ± 0.52 ^c^	0.75 ± 0.06 ^a,b^	75.56 ± 1.20 ^c^	16.02 ± 0.54 ^b,c^	89.70 ± 1.26 ^b^
SA50	0.34 ± 0.04 ^a^	25.77 ± 0.62 ^a^	0.44 ± 0.04 ^a^	9.53 ± 0.13 ^a^	0.78 ± 0.06 ^a,b^	98.65 ± 2.15 ^b^	15.66 ± 0.51 ^b,c^	98.78 ± 3.26 ^a^
EA0	0.20 ± 0.03 ^b^	20.21 ± 0.05 ^d^	0.13 ± 0.02 ^e^	1.28 ± 0.16 ^f^	0.66 ± 0.07 ^d^	40.66 ± 0.89 ^e^	17.63 ± 0.53 ^a^	70.77 ± 0.84 ^d,e^
EA10	0.30 ± 0.05 ^a^	21.52 ± 0.78 ^c,d^	0.19 ± 0.01 ^d^	5.45 ± 0.14 ^d^	0.79 ± 0.02 ^a,b^	54.11 ± 2.72 ^d^	17.17 ± 0.23 ^a,b^	76.25 ± 2.40 ^c,d^
EA30	0.35 ± 0.01 ^a^	23.75 ± 0.33 ^b^	0.30 ± 0.01 ^c^	7.20 ± 0.09 ^c^	0.81 ± 0.01 ^a^	78.24 ± 3.12 ^c^	17.17 ± 0.12 ^c^	86.95 ± 0.93 ^b^
EA50	0.36 ± 0.04 ^a^	25.34 ± 0.50 ^a,b^	0.37 ± 0.01 ^b^	8.75 ± 0.16 ^b^	0.71 ± 0.03 ^c,d^	110.58 ± 1.27 ^a^	15.36 ± 1.03 ^c^	96.43 ± 1.73 ^a^
WA	0.69 ± 0.01	50.98 ± 1.31	1.26 ± 0.02	20.45 ± 0.40	0.69 ± 0.01	185.45 ± 4.23	6.69 ± 0.25	229.24 ± 0.89

All data are the mean of three measurements ± standard deviation. Values followed by the same letter within a column are not significantly different (*p* < 0.05; Tukey’s test); designation of cake variants as in Table 2; WA were not considered in the statistical analysis.

**Table 5:** Bioactive compounds identified by UPLC-PDA-Q/TOF-MS in shortcrust pastries containing chokeberry pomace.

Group of Polyphenols	Compound	*t*_R_ (min)	λ_max_ (nm)	[M − H]^−^ (*m*/*z*) ^a^	
				MS	MS/MS
Anthocyanins	Cyanidin-3-*O*-galactoside	4.241	516	449+	287
	Cyanidin-3-*O*-glucoside	4.530	517	449+	287
	Cyanidin-3-*O*-arabinoside	4.754	515	419+	287
	Cyanidin-3-*O*-xyloside	5.419	515	419+	287
Flavonols	Quercetin-3-*O*-robinobioside	6.103	353	609	463/301
	Quercetin-3-*O*-rutinoside	6.400	353	609	463/301
	Quercetin-3-*O*-galactoside	6.532	352	463	301
	Quercetin-3-*O*-glucoside	6.593	352	463	301
	Derivative of quercetin	6.743	-	-	-
	Eriodictyol-glucuronide	6.892	280	463	287
Phenolic acids	Neochlorogenic acid	2.983	323	353	191
	Cryptochlorogenic acid	3.727	323	353	191
	Chlorogenic acid	3.968	323	353	191
Flavan-3-ols	Procyanidin B2	4.989	278	577	289

^a^ [M + H]^+^ (*m*/*z*) for anthocyanins were obtained in the positive ion mode.

**Table 6:** Polyphenol concentration (mg/kg) in shortcrust pastries containing chokeberry pomace determined by UPLC-PDA-FL *.

Cake Variant	Phenolic Compounds (mg/kg)														Total Phenolic Compounds
	Anthocyanins				Flavonols					Phenolic Acids			Flavan-3-ols		
	Cyanidin-3-*O*-Galactoside	Cyanidin-3-*O*-Glucoside	Cyanidin-3-*O*-Arabinoside	Cyanidin-3-*O*-Xyloside	Quercetin-3-*O*-Robinobioside	Quercetin-3-*O*-Rutinoside	Quercetin-3-*O*-Galactoside	Quercetin-3-*O*-Glucoside	Derivative of Quercetin	Neochlorogenic Acid	Cryptochlorogenic Acid	Chlorogenic Acid	Procyanidin B2	Eriodictyol-Glucuronide	
SA0	nd	nd	nd	nd	nd	nd	nd	nd	nd	nd	nd	nd	nd	nd	nd
SA10	52.56 ± 10.66 ^d^	2.27 ± 0.28 ^d^	20.70 ± 4.52 ^d^	2.98 ± 0.65 ^d^	2.06 ± 0.67 ^c^	2.37 ± 0.45 ^c^	0.42 ± 0.07 ^c^	6.21 ± 1.42 ^c^	4.24 ± 0.80 ^c^	10.59 ± 1.32 ^c^	0.25 ± 0.13 ^c^	29.10 ± 4.26 ^c^	5.96 ± 0.30 ^c^	8.55 ± 1.85 ^c^	148.3 ± 26.8 ^c^
SA30	120.40 ± 2.56 ^c^	4.92 ± 0.18 ^c^	46.33 ± 1.67 ^c^	5.47 ± 0.57 ^c^	6.59 ± 0.05 ^b^	6.55 ± 0.11 ^b^	1.16 ± 0.00 ^b^	15.90 ± 0.20 ^b^	11.28 ± 0.10 ^b^	34.37 ± 0.39 ^b^	0.95 ± 0.00 ^b^	93.90 ± 0.84 ^b^	18.12 ± 0.42 ^b^	32.71 ± 1.32 ^b^	398.6 ± 3.4 ^b^
SA50	200.83 ± 17.99 ^b^	9.31 ± 0.49 ^b^	77.47 ± 6.36 ^b^	9.48 ± 1.01 ^b^	13.31 ± 1.41 ^a^	13.54 ± 1.41 ^a^	2.43 ± 0.23 ^a^	32.65 ± 3.87 ^a^	23.52 ± 2.33 ^a^	72.60 ± 4.88 ^a^	1.85 ± 0.09 ^a^	202.43 ± 15.49 ^a^	37.43 ± 3.75 ^a^	78.68 ± 1.43 ^a^	775.5 ± 57.9 ^a^
EA0	nd	nd	nd	nd	nd	nd	nd	nd	nd	nd	nd	nd	nd	nd	nd
EA10	44.72 ± 3.14 ^d^	1.60 ± 0.53 ^d^	17.47 ± 1.48 ^d^	1.65 ± 0.01 ^d^	1.79 ± 0.24 ^c^	2.18 ± 0.47 ^c^	0.36 ± 0.05 ^c^	4.69 ± 0.42 ^c^	3.33 ± 0.07 ^c^	9.68 ± 0.06 ^c^	0.27 ± 0.02 ^c^	26.35 ± 0.94 ^c^	7.20 ± 0.11 ^c^	5.75 ± 1.65 ^c^	127.0 ± 5.7 ^c^
EA30	139.51 ± 5.48 ^c^	6.13 ± 0.28 ^c^	55.21 ± 3.11 ^c^	6.56 ± 0.16 ^c^	6.10 ± 0.23 ^b^	6.42 ± 0.17 ^b^	1.26 ± 0.03 ^b^	16.00 ± 0.53 ^b^	11.15 ± 0.35 ^b^	33.49 ± 0.36 ^b^	0.89 ± 0.00 ^b^	90.82 ± 1.47 ^b^	15.03 ± 0.52 ^b^	37.23 ± 1.62 ^b^	425.8 ± 13.3 ^b^
EA50	238.13 ± 44.50 ^a^	11.12 ± 2.10 ^a^	95.38 ± 19.33 ^a^	12.13 ± 2.23 ^a^	11.17 ± 2.06 ^a^	11.52 ± 2.32 ^a^	2.36 ± 0.57 ^a^	30.52 ± 7.13 ^a^	22.09 ± 4.39 ^a^	68.60 ± 11.99 ^a^	1.58 ± 0.23 ^a^	185.15 ± 33.07 ^a^	35.14 ± 2.70 ^a^	82.71 ± 17.04 ^a^	807.6 ± 149.6 ^a^
WA	1090.29 ± 15.15	55.66 ± 2.52	498.72 ± 1.14	66.35 ± 4.99	46.51 ± 0.19	46.96 ± 1.73	8.98 ± 0.33	118.42 ± 0.60	88.61 ± 1.15	369.24 ± 6.58	9.53 ± 2.39	953.43 ± 16.76	415.33 ± 26.09	373.37 ± 17.43	4141.4 ± 20.4

* UPLC-PDA-FL—ultra performance liquid chromatography with photodiode-array, and fluorescence detectors. All data are the mean of three measurements ± standard deviation. Values followed by the same letter within a column are not significantly different (*p* < 0.05; Tukey’s test); designation of cake variants as in Table 2; nd—not detected; WA were not considered in the statistical analysis.

**Table 7:** Antioxidant (ABTS, ORAC) and anti-diabetic (anti-α-amylase, anti-α-glucosidase and anti-lipase) properties of shortcrust pastries containing chokeberry pomace.

Cake Variant	Antioxidant Activity (mmoL TE/100 g)		Enzyme Inhibition IC_50_ (mg/mL)		
	ABTS	ORAC	Anti-α-Amylase	Anti-α-Glucosidase	Anti-Lipase
SA0	0.032 ± 0.004 ^d^	1.153 ± 0.054 ^f^	221.76^0.9643^ *^,d^	4454.54^0.6266^ *^,d^	16.28^0.9952^ *^,d^
SA10	0.403 ± 0.003 ^d^	2.165 ± 0.040 ^d^	18.57^0.9998^ *^,b^	1122.22^0.8103^ *^,b^	10.77^0.9948^ *^,a,b^
SA30	2.454 ± 0.086 ^b^	5.113 ± 0.106 ^b^	1.37^0.9368^ *^,a^	336.21^0.5931^ *^,a^	10.37^0.9870^ *^,a,b^
SA50	2.811 ± 0.189 ^a,b^	6.326 ± 0.194 ^a^	<0.50^0.9643^ *^,a^	178.23^0.9633^ *^,a^	9.12^0.9822^ *^,a^
EA0	0.022 ± 0.001 ^d^	1.138 ± 0.048 ^f^	202.32^0.9899^ *^,c^	3758.39^0.7095^ *^,c^	16.76^0.9820^ *^,d^
EA10	0.330 ± 0.006 ^d^	1.850 ± 0.039 ^e^	5.11^0.9910^ *^,a,b^	949.33^0.9633^ *^,b^	12.82^0.9599^ *^,c^
EA30	1.626 ± 0.106 ^c^	4.138 ± 0.123 ^c^	<0.50^0.9896^ *^,a^	934.03^0.8812^ *^,b^	12.07^0.9764^ *^,b,c^
EA50	2.911 ± 0.363 ^a^	6.385 ± 0.104 ^a^	<0.50^0.9726^ *^,a^	393.32^0.9693^ *^,a^	9.97^0.9915^ *^,a^
WA	19.742 ± 0.802	26.022 ± 0.260	<0.50^0.9992^ *	<0.10^0.9990^ *	1.62^0.9961^ *

All data are the mean of three measurements ± standard deviation (ABTS, ORAC). Values followed by the same letter within a column are not significantly different (*p* < 0.05; Tukey’s test); TE—Trolox; * R value (anti-α-amylase, anti-α-glucosidase, anti-lipase); designation of cake variants as in Table 2; WA were not considered in the statistical analysis.

## Data Availability

All the data are reported in the article.
